# Force From Filaments: The Role of the Cytoskeleton and Extracellular Matrix in the Gating of Mechanosensitive Channels

**DOI:** 10.3389/fcell.2022.886048

**Published:** 2022-05-02

**Authors:** Yu-Chia Chuang, Chih-Cheng Chen

**Affiliations:** ^1^ Institute of Biomedical Sciences, Academia Sinica, Taipei, Taiwan; ^2^ Neuroscience Program of Academia Sinica, Academia Sinica, Taipei, Taiwan; ^3^ Taiwan Mouse Clinic, BioTReC, Academia Sinica, Taipei, Taiwan

**Keywords:** mechanotransduction, ion channel, ASIC, Piezo2, ultrasound, ASIC3, PDMS, TRPA1

## Abstract

The senses of proprioception, touch, hearing, and blood pressure on mechanosensitive ion channels that transduce mechanical stimuli with high sensitivity and speed. This conversion process is usually called mechanotransduction. From nematode MEC-4/10 to mammalian PIEZO1/2, mechanosensitive ion channels have evolved into several protein families that use variant gating models to convert different forms of mechanical force into electrical signals. In addition to the model of channel gating by stretching from lipid bilayers, another potent model is the opening of channels by force tethering: a membrane-bound channel is elastically tethered directly or indirectly between the cytoskeleton and the extracellular molecules, and the tethering molecules convey force to change the channel structure into an activation form. In general, the mechanical stimulation forces the extracellular structure to move relative to the cytoskeleton, deforming the most compliant component in the system that serves as a gating spring. Here we review recent studies focusing on the ion channel mechanically activated by a tethering force, the mechanotransduction-involved cytoskeletal protein, and the extracellular matrix. The mechanosensitive channel PIEZO2, DEG/ENaC family proteins such as acid-sensing ion channels, and transient receptor potential family members such as NompC are discussed. State-of-the-art techniques, such as polydimethylsiloxane indentation, the pillar array, and micropipette-guided ultrasound stimulation, which are beneficial tools for exploring the tether model, are also discussed.

## Introduction

All organisms possess the ability to sense mechanical or physical cues or similar cues. Creatures’ exploration, communication, and survival rely on mechanosensation to recognize external and internal forces. All cells in our bodies are active components sensing mechanical inputs and performing biological responses. Differing from other senses, such as sight, smell, and taste initiated by the lock-and-key binding of ligands to G-protein-coupled receptors, the molecular basis of mechanosensation remains largely unknown.

According to the hypothesis of solute and solvent (Kung, 2005), as compared with perceiving the signals from ligands (solutes), the sensation of mechanical forces such as touch, vibration, hearing, and osmolality results from perceiving a change in the environment, which consists of water (the solvent). Thus, different from the lock-and-key binding between ligands and receptors, cell membranes are equipped with specific ion channels recognizing pressure stimuli from surrounding water. This situation assumes that the responses of cell membranes to osmotic and other mechanical forces that can stretch lipid bilayers is likely among the first senses to deal with the primitive sea that jeopardizes their membranes (Anishkin et al., 2014). Because all cells have to deal with osmotic force, knowing how membranes equipped with mechanosensitive (MS) ion channels to respond the surrounding concentration of water may be key to understanding the molecular mechanisms of mechanosensation (Kung, 2005). To navigate the environment, beyond sensing the osmotic forces, animals have evolved specialized sensory cells to convert the mechanical signals into electrical ones to be processed by the nervous system. This process, called mechanotransduction, constitutes the essential basis of force detection. Mechanotransduction is generated from the interface consisting of lipids and rigid elements perceiving the force to activate the MS ion channels. The complexity of the lipid-facing surface that transduces forces has increased during evolution. In addition to the cell wall and the membrane of the lipid bilayer, extracellular matrix (ECM), cytoskeleton, and MS-associated proteins are critical to the gating of MS ion channels to convert mechanical stimuli into ion flux.

In distinct cell types, different types of mechanical inputs lead to the activation of sensors by specialized mechanisms. Many studies have focused on mechanotransduction in cochlear hair cells, lung, kidney, bladder, and somatosensory neurons such as dorsal root ganglia (DRG). Several vital MS ion channels have been successively revealed, and varied neurological diseases in humans were found associated with mutations in MS channels. A breakthrough finding in 2010 established PIEZO1 and PIEZO2 as *bona fide* MS cation channels (Coste et al., 2010). Subsequent studies have revealed new roles of PIEZOs in touch, mechanical pain, itch, proprioception, breathing, gastrointestinal tract, bladder, baroreflex, hearing, cartilage, bone development, etc. (Murthy et al., 2017; Szczot et al., 2021). Mutations in PIEZO1 and PIEZO2 are linked to multiple hereditary human diseases (Volkers et al., 2015; Alper, 2017). Loss-of-function mutations in human PIEZO1 results in autosomal recessive congenital lymphatic dysplasia (Lukacs et al., 2015), and gain-of-function mutations of PIEZO1 cause the disease dehydrated hereditary stomatocytosis (Zarychanski et al., 2012; Albuisson et al., 2013; Andolfo et al., 2013). Loss-of-function mutations in human PIEZO2 results in profoundly decreased proprioception leading to ataxia and dysmetria (Chesler et al., 2016), and gain-of-function mutations of PIEZO2 are directly linked to distal arthrogyposis type 5, Gordon syndrome, and Marden-Walker syndrome (Coste et al., 2013; McMillin et al., 2014). Transient receptor potential (TRP) channels are polymodal and activated by various stimuli including temperature, voltage, small molecules, and mechanical forces (Árnadóttir and Chalfie, 2010). For example, TRPV4 is required for the proper response to hypotonic and hypertonic stimuli in nociceptive fibers of rodents (Alessandri-Haber et al., 2003; Alessandri-Haber et al., 2005). Mutations in TRPV4 have been implicated in skeleton and nervous system diseases: familial digital arthropathy-brachydactyly (Lamandé et al., 2011), Charcot–Marie–Tooth disease 2C (Deng et al., 2010; Landouré et al., 2010), and scapuloperoneal spinal muscular atrophy (Auer-Grumbach et al., 2010; Zimon et al., 2010). Despite a clear association between mutations of specific MS channels and disease, the exact roles of mechanosensation mediated by MS channels in an array of physiological and pathological processes are enigmatic. Therefore, uncovering the modulation and gating mechanism of MS ion channels represents a fundamental challenge to help better understand the physiological function and pathogenic mechanisms of mechanosensation.

In terms of the gating mechanism, two models have been proposed to illustrate how mechanical force activates MS channels. One is the model of the “force-from-lipids” (the membrane tension model), which is the force applied to the lipid bilayer directly to switch the MS channel to an open conformation (Martinac et al., 1990; Kung, 2005; Cox et al., 2017). In 1990, Martinac et al. (1990) found that applied amphipathic compounds can increase the membrane tension to gate bacterial MS channels in giant spheroplasts of *Escherichia coli* and first proposed the force-from-lipids model. About 4 years later, Sukharev et al. (1994), Häse et al. (1995) reconstituted purified MscL, a large mechanosensitive ion channel of *E. coli*, into lipid bilayers to examine the function by the patch-clamp technique and showed that the force-from-lipids was sufficient for the activation of MS channels by mechanical force. The other model is “force-from-filament” or “force-from-tether” (the tether model). Force applied to the ECM or extracellular accessory proteins is transmitted through a tether directly or indirectly connecting the channel with the cytoskeleton or cytoskeleton-associated proteins to gate the channel (Katta et al., 2015; Ranade et al., 2015; Zhang et al., 2015). As compared with the multiple factors involved in the tether model, the membrane tension model is relatively straightforward and uncomplicated. A reductionist view indicated that the gating of MS channels can be reduced to fit the force-from-lipids principle (Cox et al., 2017). Considering that conformation change is required for the gating of ion channels, Cox et al. (2017) created an ion channel mechanical continuum whereby all ion channels can be placed from highly sensitive genes such as PIEZO1 to genes insensitive to applied force such as KcsA. Therefore, highly sensitive MS channels can be activated by low mechanical stress applied to the lipid bilayers. In this hypothesis, the position of the ion channels on the mechanical continuum can be shifted by the specific cellular localization and the interaction with structural scaffold proteins or auxiliary proteins that can share or focalize the force. The paradigm of force-from-filament is used to explain how the tethering molecules modulate the sensitivity and extend the dynamic range in this mechanical continuum. The models of the force-from-lipids and force-from-filament may not be mutually exclusive. One should not consider these two paradigms as an absolute dichotomy because mechanosensory transduction is generated by a highly dynamic interaction between the lipid bilayers, membrane-anchoring scaffold proteins, cytoskeleton, and ECM (Kung, 2005; Cox et al., 2017; Cox et al., 2019). Therefore, the force-sharing model has been proposed to properly depict the gating mechanism (Chalfie, 2009; Martinac, 2014; Cox et al., 2019). For example, a battery of information about PIEZO1 has demonstrated that both cytoskeletal proteins and ECM molecules can dynamically modulate the activity of inherently MS bilayer-gated channels (Cox et al., 2016; Bavi et al., 2019). However, these paradigms are useful to help explore novel MS channels and better understand how mechanical force is distributed between distinct components required for MS channel gating.

The necessary and sufficient criteria for defining an authentic MS channel have been developed. Four criteria have been proposed and are generally accepted to classify MS channels (Árnadóttir and Chalfie, 2010; Ridone et al., 2019; Nicoletti et al., 2021): First, the channel must be expressed temporally and spatially within the mechanosensory organ and should not be necessary for cell maturation or integrity. Second, the ion channel must be critical for mechanosensitivity, and its removal abolishes the ability of the cell to respond to mechanical stimulation. Third, structural mutations that alter the properties of the channel protein should alter the properties of the mechanical responses. This criterion supports a direct role for the protein in mechanotransduction. Fourth, heterologous expression of the channel protein should result in robust mechanosensory responses in the host cell. For the MS channels requiring a tethering force to be gated, the last two criteria are difficult to meet. In the third criterion, because multiple auxiliary subunits could adapt the biophysical properties of the channel, in some conditions, this does not guarantee that the considered channel is MS. In the fourth criterion, because of the requirement of auxiliary subunits such as specific ECM- and cytoskeleton-associated proteins, the pore-forming subunits gated by force-from-filament to fit the criterion is challenging. From ENaC/DEG-superfamily to PIEZO1/2, a series of reviews have summarized the detailed information about the identified or confirmed MS ion channels, covering the biophysical properties of the structure to the biological function to show how the specific mechanotransduction regulates and balances the physiologic conditions (Chalfie, 2009; Árnadóttir and Chalfie, 2010; Katta et al., 2015; Ranade et al., 2015; Martino et al., 2018; Jin et al., 2020; Nicoletti et al., 2021; Szczot et al., 2021). However, there is not much discussion on the auxiliary subunits involved in the force-from-filament. In this review, we aim to provide a brief overview of the current knowledge on the potential mechanism of neuronal mechanosensitivity involving connections among the cytoskeleton, ECM, and MS channels. We discuss state-of-the-art techniques for dissection the tethering model, including the elastic substrate, the pillar array, and ultrasound. The MS channels reviewed are summarized in Table 1.

**TABLE 1:** Evidence supporting force-from-tether gating of putative mechanosensitive ion channels.

Channel	Gating condition	Involved cytoskeleton and ECM	Stimulation form	Ref
PIEZO				
PIEZO1	Activated for Ca^2+^ influx by matrigel or collagen IV-mediated pulling force*	ECM: collagen IV (no evidence shows direct interaction)	AFM cantilevers coated	Gaub and Müller, (2017)
	Sensitization of PIEZO1 mediated by nonmuscle myosin II-dependent actin contractility*	Actin filaments (cytochalasin D) NM mysoin II (blebbistatin)	Elastomeric pillar arrays, whole-cell recording with pillar deflection	Bavi et al. (2019)
PIEZO2	Latrunculin A increased the threshold for activation*	Actin filaments (latrunculin A)	Whole-cell recording with PIEZO-driven glass probe stimulation	Eijkelkamp et al. (2013)
	Colchicine reduced hPIEZO2 peak currents, but did not increase activation threshold*	Microtubules (colchicine)	Whole-cell recording with PIEZO-driven glass probe stimulation	Eijkelkamp et al. (2013)
	Latrunculin A enhanced margaric acid-mediated PIEZO2 inhibition*	Actin filaments (latrunculin A)	Whole-cell recording with PIEZO-driven glass probe stimulation	Romero et al. (2020)
	Cytochalasin-D reduced current amplitudes, but did not alter activation threshold and inactivation kinetics*	Actin filaments (cytochalasin D)	Whole-cell recording with PIEZO-driven glass probe stimulation	Verkest et al. (2022)
DEG/ENaC/ASICs				
MEC-4/10	ECM components required for the correct localization of MS channel complex and touch sensitivity*	ECM: MEC-1, MEC-5	Gently stroking the animal with an eyebrow hair	Emtage et al. (2004)
	15-protofilament microtubules modulating mechanotransduction but are not required for mechanogating of MEC-4 complex*	Microtubules: MEC-7, MEC-12	*In vivo* whole-cell recording with piezo-driven glass probe stimulation	O'Hagan et al. (2005)
ASIC1	Gated by a combination of compression force and pulling force with low-intensity and low-pressure ultrasound*	Actin filaments (cytochalasin D) microtubules (nocodazole)	Ultrasound	Lim et al. (2021)
ASIC3	Gated by ECM-coated substrate stretching, not by cell membrane indentation*	Actin filaments (cytochalasin D) microtubules (nocodazole) ECM: fibronectin	Indentation on PDMS	Lin et al. (2009), Lin et al. (2016)
ENaC	N-glycosylation mediated gating with intact ECM environment*	ECM: N-linked glycans	TEVC recordings or whole-cell recordings with shear force	Knoepp et al. (2018b)
TRP				
NompC	ARD functioning as a tether linking with microtubules	Microtubules (nocodazole, colcemid)	Inside-out patch with pressure clamp, whole-cell recording with PIEZO-driven glass probe stimulation	Zhang et al. (2015)
TRPA1	Gated by 7 MHz focused ultrasound with intact cytoskeleton*	Actin filaments (cytochalasin D, latrunculin A, jasplakinolide)	Whole-cell recording with ultrasound	Duque et al. (2022)
TRPV-1	Push activation by microtubule interaction*	Microtubule (nocodazole, paclitaxel)	Whole-cell recording with osmotically induced shrinking, single channel recording with pressure-clamp	Prager-Khoutorsky et al. (2014)
TRPV4	Activated by deflection of cell-substrate contacts points, but not by membrane stretching*	ECM	Elastomeric pillar arrays, whole-cell recording with press clamp	Servin-Vences et al. (2017)
Others				
The complex of TMC-1/2, TMIE, and LHFPL5	Receiving force from tip links	Tip link (extracellular filaments)	Whole-cell patch with hair bundle deflection	Maeda et al. (2014), Zhao et al. (2014), Ge et al. (2018)

^*^No direct evidence for tethering model.

## Force From Filament, the Tether Model of Mechanosensitive Ion Channels

In the model of the force-from-filament, the cytoskeleton and/or ECM proteins connect to MS ion channels, directly or indirectly transmitting the force to activate the MS channels. The tethering filaments may directly interact with the channel proteins or anchor to auxiliary proteins, indirectly imposing tension on the channels (Cox et al., 2019). Mechanotransduction in mammalian hair cells of the auditory system has been the most well studied for its tethered mechanisms (Marcovich and Holt, 2020; Nicoletti et al., 2021). The stereocilia tip of hair cells converts mechanical forces into electrical signals *via* a mechano-electrical transduction (MeT) machinery formed by stretch-gated ionic channels and tip-link filaments (Cunningham and Müller, 2019). There are three candidates of MeT channels, most fulfilling the criteria of pore-forming MS channels, including lipoma high mobility group IC fusion partner-like 5 (LHFPL5)/tetraspan membrane protein of hair cell stereocilia (TMHS), transmembrane inner ear (TMIE), and transmembrane channel-like proteins 1 and 2 (TMC1/2) (Cunningham and Müller, 2019). PIEZO2, the MS channel refractory to activation by membrane stretch (Cox et al., 2019; Zheng et al., 2019), is not essential for auditory function, although it is expressed in hair cells (Wu et al., 2017a).

In a report by Syeda et al. (2016) PIEZO1 was found activated by the force-from-lipids in experiments using purified protein reconstituted in a lipid environment. Although PIEZO1 is inherently MS without additional cellular components for activation, its activation is tuned by multiple mechanisms. For example, an experiment using atomic force microscopy-based assay to mechanically stimulate PIEZO1 receptors in living human and mouse cells demonstrated that ECM components were critical for mechanical activation of PIEZO1 (Gaub and Müller, 2017). Bavi et al. (2019) showed evidence of the force from substrate deflections affecting membrane tension and cytoskeleton stress, both activating and regulating PIEZO1. Of note, accumulating data has indicated that actomyosin-dependent contractility can sensitize both outside-in signaling (Bavi et al., 2019) and inside-out signaling (Pathak et al., 2014; Ellefsen et al., 2019) *via* PIEZO1 within the cell–substrate interface. In contrast, PIEZO2 is intriguingly known for a preferential response to indentation of the cell membrane, whereas PIEZO1 is sensitive to all forms of mechanical deformation (Ikeda and Gu, 2014; Moroni et al., 2018; Shin et al., 2019). A battery of data has indicated PIEZO2 is less sensitive to membrane stretch (Coste et al., 2015; Moroni et al., 2018; Verkest et al., 2022). Shin et al. (2019) showed that positive pressures under the cell-attached mode could activate PIEZO2 currents in a Merkel cell line and PIEZO2-overexpressed HEK 293T cells, but negative pressure failed to activate PIEZO2 in these cells, which suggests that not all kinds of membrane stretching can induce PIEZO2 gating. Similarly, Moroni et al. (2018) could not record the stretch-activated currents in excised or cell-attached patches from PIEZO2-overexpressed N2a^PIEZO1−/−^ cells. Although PIEZO2 and PIEZO1 have been found sensitized by the integral membrane protein STOML3, which may increase membrane stiffness to facilitate force transmission, PIEZO2 may apply a distinct mechanism from PIEZO1 to the channel gating (Wu et al., 2017b). Even though the mechanism of the force-from-tether remains an appealing possibility, there is little evidence to establish the comprehensive gating model. In a recent report, Wang et al. (2022) indicated that PIEZO channels interact with the complex of cadherin, beta-catenin, and F-actin. The authors found critical mechanogating domains of PIEZO1 for E-cadherin binding that disrupted the interaction between PIEZO1 and E-cadherin, thus preventing F-actin dependent gating. This solid evidence suggests that PIEZO channels are biochemically and functionally tethered to the actin cytoskeleton *via* the cadherin mechanotransduction complex.

NompC, a member of the TRPN subfamily of ion channels, is expressed in *Drosophila* peripheral sensory organs, essential for mechanosensory responses, and is involved in mechanosensory-related behaviors (Walker et al., 2000). In the view of channel structure for tethering, the unique structure of NompC mainly consists of a gigantic ankyrin repeat domain (ARD) in the cytosolic part (Jin et al., 2020) that functions as a linker associated with microtubule proteins (Cheng et al., 2010). Consistent with the tether model, the ARD of NompC acts as a spring that transmits the movement of the microtubules for the channel opening. Although we do not know how the microtubule protein interacts with the ARD to transduce force, a collection of observations revealed that NompC physically interacts with microtubules, which suggests that the microtubule is required for NompC activation (Cheng et al., 2010; Zhang et al., 2015; Yan et al., 2018). NompC is a *bona fide* mechanically activated ion channel, but there are no mammalian homologs (Yan et al., 2013).

The gating mechanism of the TRP superfamily is still debated (Nicoletti et al., 2021). In neuronal cells of mammals, TRPV-1/2/4 are involved in the mechanosensation of osmosensory neurons, mechanosensory neurons, and primate retinal ganglion cells (Mihara et al., 2010; Prager-Khoutorsky et al., 2014; Gao et al., 2019). TRPC-1/3/5/6 mediate light touch and sound responses (Quick et al., 2012; Sexton et al., 2016). TRPA1 contributes to mechanical nociception (Kwan et al., 2006). Maroto et al. (2005) indicated that TRPC-1 is gated by tension developed in the lipid bilayer. In contrast, the interaction between microtubules and TRPV1 was found required for the osmosensation (Prager-Khoutorsky et al., 2014). Nikolaev et al. (2019) tested stretch activation of 11 TRP channels from six mammalian subfamilies and found that the mammalian members of the TRP ion channel family are insensitive to tension induced by cell membrane stretching. Therefore, TRP channels are more likely to be activated by cytoplasmic tethers or downstream components as amplifiers of mechanosensory signaling cascades. In terms of structure, two groups applied full-atom molecular dynamics simulations and force-balance analysis to NompC, revealing that compression of the ARDs can produce a rotational torque on the TRP domain (Argudo et al., 2019; Wang et al., 2021). The observations provide a plausible push-to-open mechanism for the tethered ion channel NompC (Wang et al., 2021), similar to the TRPV1 push-to-open gating transition (Yang et al., 2018), which suggests a universal gating mechanism of the TRP channel to feel compression and shrinkage. In contrast, Deng et al. (2018) presented the cryo-EM and X-ray diffraction structure of *Xenopus tropicalis* TRPV4, and unique structural properties provided few clues implying its putative gating by mechanical stimuli. In addition, Servin-Vences et al. (2017) used the pillar system to demonstrate that only TRPV4 can be activated by stimuli applied at cell–substrate contact, whereas PIEZO1 can generate a current by cell membrane stretching. However, this observation could not exclude that the actin cytoskeleton functions as a mechanoprotectant to inhibit stretch activation of TRPV4. Most important, recent data have shown that the activation of TRPV4 by pillar deflection is not influenced by disruption of the actin cytoskeleton (Sianati et al., 2020). The role of the cytoskeleton in tethering-gated TRP channels still needs to be elucidated.

Ion channels of the degenerin/epithelium sodium channel/acid-sensing ion channel (DEG/ENaC/ASIC) superfamily are another representative MS channels gated by the force-from-filament. ENaC responds to shear forces in endothelial cells, crucial for blood pressure regulation (Knoepp et al., 2018a; Knoepp et al., 2018b). The pore-forming α subunit of ENaC is in direct contact with the ECM/glycocalyx *via* two N-glycosylated asparagines in the palm and knuckle domains (Knoepp et al., 2018b). Removal or alteration of the ECM-contacting structure decreased shearing force-induced ENaC currents. The evidence, in agreement with the force-from-filament paradigm, establishes a connection to the ECM that facilitates vascular responsiveness contributing to blood-pressure regulation (Knoepp et al., 2020). Of note, ENaC is also activated by Cys-palmitoylation (Mueller et al., 2010; Mukherjee et al., 2014; Kleyman and Eaton, 2020). The transmission of mechanical force *via* a covalently bound lipid may be a form of force-from-lipids (Cox et al., 2019). However, these observations suggest that post-translational modification could be essential to the mechanotransduction of some MS channels and might hinder the explanation of data shown in heterologous expression systems.

Among the DEG/ENaC/ASIC channels, the most-studied MS channels are *Caenorhabditis elegans* MEC-4 and MEC-10, which are expressed in mechanosensory neurons responsible for touch sensation. Chalfie and colleagues conducted phenotyping studies of loss-of-function or gain-of-function mutations in worm genes, which led elegantly to identifying a series of mechanosensitivity genes (the *mec* genes) (Ernstrom and Chalfie, 2002; Goodman and Schwarz, 2003; Bianchi et al., 2004). The bundle of MEC genes has been proposed to construct a convincing model of the force-from-filament (Chalfie, 2009). In this blueprint, MEC-4 and MEC-10, as the pore-forming subunits, assemble in homo- or hetero-trimers. The model illustrates that MEC-4/MEC-10 interacts with the extracellular proteins MEC-1, MEC-5 (a neuron-specific collagen), and MEC-9 and with the cytoskeletal tubulins MEC-7 (beta-tubulin) and MEC-12 (alpha-tubulin) (Hamelin et al., 1992; Du et al., 1996; Fukushige et al., 1999; Emtage et al., 2004). However, we lack evidence to show the *in vivo* co-localization of these clusters (Cueva et al., 2007), and removal of the special tubules had only limited effect on the structure or function of these clusters (Emtage et al., 2004; Zhang et al., 2004). Although these findings cast doubt on whether the nematode MS channel is directly gated by microtubule tethering, the interaction of MEC-4/MEC-10 with the stomatin-related protein MEC-2 is critical for the channel activity (Goodman et al., 2002; Zhang et al., 2004; Brown et al., 2008). The MEC-2 protein is a membrane scaffolding protein belonging to the stomatin-like protein family. The central stomatin-like domain is predominantly palmitoylated and associated with lipid rafts. In terms of the mammalian homologs of MEC-2, stomatin-like protein 3 (STOML3) has been convincingly found necessary for normal touch transduction in mice. In isolated DRG neurons from Stoml3-knockout mice, a population of neurons was less responsive to the neurite indentation (Wetzel et al., 2007). Furthermore, STOML3 facilitates force transfer to ion channels, such as PIEZO1, mediated by neuron–substrate adhesion domains, which suggests the potential role of extracellular tethering (Poole et al., 2014).

ASICs have been proposed as mechanical transducers involved in neuronal mechanotransduction by evidence from genetic studies, although the mechanical gating mechanism is still unclear (Chen and Wong, 2013). Because of the tethering model established in the MEC complex, the force-from-filament may activate the gating of ASIC channels. The stomatin-domain proteins, including STOML1 and STOML3, interact with ASICs and have different inhibitory effects on acid-induced currents of ASIC1a, ASIC2a, and ASIC3 (Hu et al., 2010; Moshourab et al., 2013; Omerbasic et al., 2015). *In vivo* studies showed that in Stoml3-knockout mice, about 40% of skin mechanoreceptors were less sensitive to mechanical stimuli (Wetzel et al., 2007). Because STOML3 is also a potent modulator of PIEZO1 and PIEZO2 channels that tunes the sensitivity of mechanical gating and mediates the detection of molecular-scale stimuli for fine touch (Poole et al., 2014), the role of STOML3 in the model of tethering-gated ASICs is inconclusive. To demonstrate whether ASIC3 is involved in mechanosensation, Lin and colleagues developed a tethering-mediated system to probe the mechanoresponse by indirectly stretching a single neurite of a DRG neuron (Lin et al., 2009; Lin et al., 2016). The elastic material coated with ECM/fibronectin was used for substrate deformation-driven neurite stretch (SDNS). The indentation of the substrate provides a form of mechanical stimuli mediated by ECM tethering (Lin et al., 2009; Lin et al., 2016). Thus, SDNS generates a different modality of mechanical force from the direct probe indentation on the cell membrane used in most previous studies. In contrast to ASIC3 ablation not affecting the response by direct neurite indentation, genetic ablation or pharmacological inhibition of ASIC3 in proprioceptors significantly hampered the response induced by SDNS. Thus, the deformation of the cell membrane by itself was not sufficient to activate the ASIC3 channel, which suggests that ASIC3 may apply the force-from-filament, at least *via* ECM-tethering, to gate the channel. Further investigation needs to dissect the exact molecules involved in the mechanism of tethering gating. Are there auxiliary proteins bridging ECM and ASIC3? Are there adaptor proteins interacting with the cytoskeleton and ASIC3 for the channel activation? Is ASIC3 similar to ENaC having ECM binding domains *via* post-translational modification? Do other ASICs follow the principle of the force-from-tether to be mechanically activated? The SDNS system may provide a new avenue to explore and establish the tethering model with more details (Cheng et al., 2018).

## Techniques for Studying the Tethering Model

A well-designed technique is critical to dissect the proper channel-gating model of neurosensory mechanotransduction. Before the development of the mechano-clamp, patching with membrane stretching was used to apply positive or negative pressure through a patch pipette to direct quantification of single- or multi-ion channel activity. However, the limitation was that it could be used for only single-cell assay and was inadequate for pharmacological testing. The mechanoprotective effect of the cytoskeleton is also an issue that may cause the discrepancy between the cell-attached mechanosensitivity and direct measurement (Spassova et al., 2006; Suchyna et al., 2009; Cox et al., 2016).

Because sensory afferent endings have been inaccessible to general patch-clamp recording, the mechano-clamp technique has been established to fill the assessment gap between the properties of mechano-activated currents *in vitro* and the characteristics of mechanoreceptors *in vivo* (McCarter et al., 1999; Coste et al., 2010; Hao and Delmas, 2011). By deforming the plasma membrane of sensory neurons by using a piezo-driven mechanical probe, the MS ion channels respond to the indentation of neuronal soma (McCarter et al., 1999; Hao and Delmas, 2011). This technique was designed similar to mechanical indentation of the skin. Both use focal mechanical stimulation with positive pressure to activate MS channels. This whole-cell mechano-clamp technique is widely used to probe mechanically activated currents on dissociated sensory neurons. Coste et al. (2010) applied the mechano-clamp combined with siRNA screening of MS candidates to reveal PIEZOs, the “holy grail” of mechanosensation. However, this most popular technique, which directly applies force to the neuronal soma, has several potential drawbacks.

The first drawback is that the stimuli are not physiologically relevant to the mechanotransduction of sensory nerve terminals. One study, using the indentation probe on the neurites of cultured sensory neurons, revealed that 92% of sensory neurites possessed currents gated by submicrometer displacement stimuli (Hu and Lewin, 2006). In the following study, Lewin’s group used the indentation of neurites to demonstrate that both the ECM selectivity and cytoskeletal structure play important roles in neurosensory mechanotransduction, showing evidence of the force-from-filament (Hu et al., 2010; Chiang et al., 2011). The second drawback is that in terms of biomechanics, this approach by using glass as a culture substrate fails to address important structural-related issues because the physiologic surroundings of DRG or afferent endings are softer substrates. Most mechanosensation in the physiologic condition may not come from the direct indentation on the soma or neurites but rather occurs by substrate stretching. The direct indentation of cells cultured on glass raises the possibility of damage and most importantly, is not a proper way tp investigate the tethering model, considering that the direct displacement on the cell membrane delivers a non-specific force on the cell without the specificity of the ECM ligands. To achieve this specificity, materials with elastic modulus have been used for studies based on substrate deformation.

In 2009, Chen’s group applied polydimethylsiloxane (PDMS), coated with fibronectin, as an elastic substrate to investigate the stretch-activated mechanotransduction of mouse DRG neurites (Lin et al., 2009). Although the experiments could not exclude the involvement of glia transmitters, which may be released by the stimulation of the substrate deformation, the authors identified that the ECM and cytoskeleton were involved in the distal force-mediated mechanotransduction (Lin et al., 2009). In the following study, substrate deformation-driven neurite stretches demonstrated that ASIC3 was involved in the ECM tethering-mediated mechanotransduction in proprioceptors (Lin et al., 2016). In contrast, Lewin and Poole’s group established a distinct system of the elastic substrates for the precise movement of defined substrate areas to gate MS channels (Poole et al., 2014; Sianati et al., 2019). The investigators cultured mouse DRG neurons on the laminin-coated pillar arrays of PDMS and applied stimuli by deflecting a single pilus, which was bent by a piezo-driven nanomotor. This sophisticated system precisely controlled the substrate deformation and allowed for a defined stimulus directly at localized cell–matrix contacts. The technique provided the opportunity to study molecular-scale gating of MS channels with high resolution (Poole et al., 2014; Sianati et al., 2019). The group demonstrated that STOML3 is the potent modulator of PIEZO1 that fine-tunes the sensitivity of sensory neurons to mechanical stimuli relevant for fine touch (Poole et al., 2014). Later, Lewin and Poole’s group demonstrated that by pillar bending from cell–substrate contacts, stimuli activated PIEZO1- and TRPV4-mediated currents in chondrocytes. However, only PIEZO1 mediated stretch-activated currents (Servin-Vences et al., 2017). In the following study, the pillar system with different roughness and stiffness provided evidence that stimuli applied at contacts between the cell–substrate interface synergistically activated PIEZO1 by actin-mediated contractility and tension within the lipid bilayer (Bavi et al., 2019). Even though the method combining pillar arrays and whole-cell patch-clamp has great advantages to scrutinize the model of force-from-filament, a few studies applied this technique to dissect the interaction among the cytoskeleton, MS channel, and ECM (Poole et al., 2014; Servin-Vences et al., 2017; Wetzel et al., 2017; Bavi et al., 2019; Patkunarajah et al., 2020; Sianati et al., 2020; Richardson et al., 2021). Only PIEZO1 and TRPV4 have been found as MS ion channels activated by pillar deflection (Servin-Vences et al., 2017; Sianati et al., 2020), perhaps because the fabrication of micropillar arrays has a high technical threshold; for example, keeping PDMS pillars intact without shattering is critical for the following experiments (Sianati et al., 2019). Nonetheless, the pillar system helped identify two novel components for mechanotransduction: TMEM87a/Elkin1 and a putative MS ion channel, TACAN (Tmem120 A) (Beaulieu-Laroche et al., 2020; Patkunarajah et al., 2020). Elkin1 is critical for a PIEZO1-independent mechanoelectrical transduction pathway to regulate melanoma cell migration and cell–cell interactions (Patkunarajah et al., 2020), whereas TACAN is involved in sensing mechanical pain (Beaulieu-Laroche et al., 2020; Bonet et al., 2021). Patkunarajah et al. (2020) deleted Elkin1, thus increasing interaction forces between melanoma cells and a functional ECM molecule, laminin 511, which suggests an interaction between ECM and Elkin1-dependent mechanotransduction. In contrast, Beaulieu-Laroche’s group used the pillar system to generate TACAN-mediated MS currents, but the whole-cell indentation did not show any TACAN-dependent mechanically evoked currents (Beaulieu-Laroche et al., 2020). The authors supposed that the indentation of the cell membrane of flat cell lines is difficult to reach the high threshold required for TACAN activation without perforating the membrane. Recently identified OSCA was also found as a high-threshold MS ion channel, which can be activated by the Soma indentation (Murthy et al., 2018). However, whether TACAN is an MS ion channel is still debated (Ke et al., 2021; Niu et al., 2021; Rong et al., 2021; Xue et al., 2021). Niu et al. (2021) failed to identify MS ion channel activity of TACAN by cellular patch-recording; Rong et al. (2021), Ke et al. (2021) were unable to record TACAN-mediated currents *via* poking- or stretching in PIEZO1-knockout HEK 293 cells. Although we cannot completely exclude that TACAN might only respond to certain forms of mechanical stimuli such as the force from the cell–substrate interface, a series of structure analyses by cryo-electron microscopy revealed that TACAN is a coenzyme A-binding membrane protein with structural similarities to ELOVL fatty acid elongase, so TACAN may function as an enzyme in lipid metabolism (Ke et al., 2021; Niu et al., 2021; Rong et al., 2021; Xue et al., 2021). In contrast, Chen et al. (2022) provided evidence that a single-point mutation of the key residue Met^207^ in TACAN greatly increased membrane pressure-activated currents, and cholesterol was identified in the TACAN protein. The evidence from Chen et al. (2022) implies that the wild-type TACAN is in a closed state due to the binding of coenzyme A to block ion conduction.

Membrane stretch, indentation, and substrate deformation described above have been classically applied in a low-throughput setting. A review summarized the corresponding methods for high-throughput stimulation that may be feasible for mechanical screening of MS ion channels (Szczot et al., 2021).

## Ultrasound for the Exploration of Mechanosensitive Ion Channels

**FIGURE 1:**
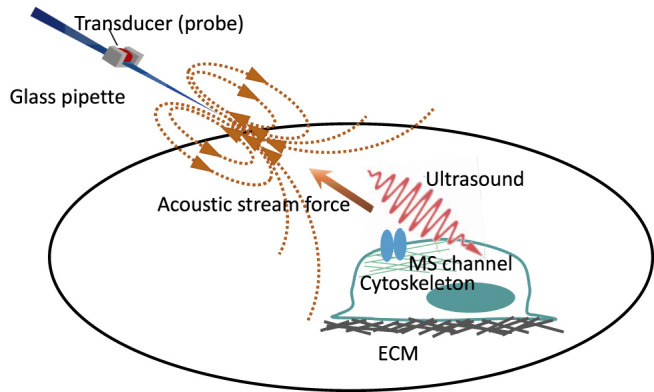
Cartoon depicting two forms of force generated by the micro-pipette-guided ultrasound. The device consists of a glass pipette and a piezoelectric transducer that functions as an ultrasound probe. The closed-ended micro-pipette will generate two relevant stimuli at the cellular level: acoustic streaming on the apical side of the cell and ultrasound propagation throughout the cell. The acoustic streaming functions as the pulling force, and the ultrasound gives the cell the compression force that may induce cytoskeletal rearrangement. Modified with permission from Lim et al. (2021) and Chu et al. (2021).

**FIGURE 2:**
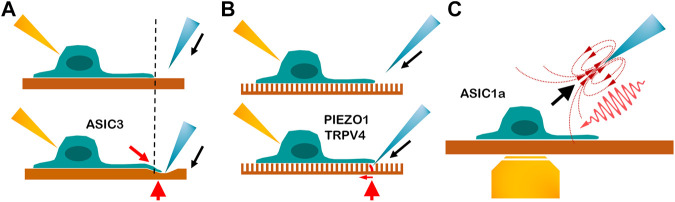
Cartoon depicting three beneficial tools for exploring the tethering model of mechanosensitive channel. **(A)** Indentation on the polydimethylsiloxane substrate. Blue is the indentation probe, yellow is the recording electrode, and red arrowhead is the tethering site. The dashed line points to the neurite stretching. The red arrow indicates the direction of the robust neurite stretching. The gating mechanism of ASIC3 is demonstrated by this tool. (Lin et al., 2016; Lin et al., 2009) **(B)** Indentation on the elastic micropillar array. The single bending pillar is labeled in red. The red arrow shows the bending direction and indicates that the force is precise and on a small scale. The pillar array demonstrates that PIEZO1 and TRPV4 can be activated by stimuli from the movement of the cell–substrate contact site. (Servin-Vences et al., 2017; Sianati et al., 2020) **(C)** Micropipette-guided ultrasound stimulation generates the pulling force (black arrow) and ultrasound compression force (red wave). Yellow is a microscope lens used for calcium imaging. Micropipette-guided ultrasound activates ASIC1a by cytoskeleton or extracellular matrix tethering Lim et al. (2021), (Chu et al., 2021).

The above methods are related to the modalities of how cells receive the steady force. Shear stress and ultrasound stimulation may be used as distinct force modalities to activate the MS ion channels. In 2021, Chu and colleagues designed a novel ultrasound probe to generate, along the propagation path, both compressional waves propagating through the cells and shear stress on cellular apical surfaces, which caused ECM and cytoskeleton combined stretching (Chu et al., 2021; Lim et al., 2021) (Figure 1). Chu et al. (2021) used a glass micropipette as a waveguide to convey ultrasound to elevate intracellular calcium level in nucleus pulposus cells. The device imposes a unique and dual loading to cell surfaces and the cytoskeleton, so it is effective in activating different mechanoreceptors, especially the type gated by the force-from-filament (Chu et al., 2021). This low-intensity ultrasound-mediated mechanotransduction (I_sppa_ = 7.4 mW/cm^2^, with streaming and 12-kPa peak pressure) was also used to activate ASIC1a with an intact cytoskeleton, which was inhibited by ASIC1a blockade and cytoskeleton-modified agents, thus suggesting that the force-from-filament may be involved in the gating of ASIC1a (Lim et al., 2021) (Figure 2). In contrast, Sorum et al. (2021) used relatively high-intensity ultrasound to activate TRAAK K^+^ channels by increasing membrane tension. The intensity of ultrasound applied to oocytes was 0.3–1.2 W/cm^2^ and to pyramidal neurons 3.6 W/cm^2^. Ye et al. (2018) used the gain-of-function mutation I92L to sensitize sonic responses of MscL and triggered channel activation at negative pressure with 0.25–0.45 MPa. Other lines of evidence showed that ultrasound can activate PIEZO1 with relative high intensity from 0.17 to 1.6 MPa (Pan et al., 2018; Liao et al., 2019; Qiu et al., 2019; Shen et al., 2021), and Prieto et al. (2018) used continuous wave ultrasound at 50 or 90 W/cm^2^ *via* cell membrane stress to activate PIEZO1. Although PIEZO1, MscL, and TRAAK are known for gating by lipid-bilayer stretching, evidence to date has revealed that only ultrasound with high peak pressure, high intensity and membrane loading form can activate these force-from-lipid gated MS channels. However, the activation of TRPA1 and ASIC1a required a modality with moderate peak pressure, low intensity, and complex loading conditions (Oh et al., 2019; Lim et al., 2021).

Miscellaneous models of ultrasound stimuli provide a variety of force modalities for *in vitro* and *in vivo* studies. Kubanek et al. (2018) found that a pulsed ultrasound stimulation evoked avoidance responses *via* MEC-4, a DEG/ENaC/ASIC ion channel. The nematode DEG channels (MEC-4 and MEC-10) were proposed to be gated by the model of force-from-tether to transduce mechanical stimuli (Chalfie, 2009). The pore-forming subunits MEC-4 and MEC-10 may interact with the ECM proteins MEC-1, MEC-5, and MEC-9 and the tubulins MEC-7 and MEC-12 (Chalfie, 2009). Two recently sonogenetic studies showed that ultrasonic neuromodulation is mediated by mechanical stress on the plasma membrane and relies on the intact organization of actin filaments (Duque et al., 2022; Yoo et al., 2022). Yoo et al. (2022) demonstrated ultrasound stimulation activating specific MS ion channels including TRPP1/2, TRPC1, and PIEZO1. Depolymerizing actin cytoskeletons of the target cells reduced the amplitude of the ultrasound-evoked calcium response. However, the findings of Yoo et al. (2022) provide only a mechanistic explanation that ultrasound activates neurons by mechanosensitive calcium accumulation; they do not indicate the specific MS ion channels that are directly gated by ultrasound force. In contrast, Duque et al. (2022) revealed that ultrasound evoking gating of hsTRPA1 specifically requires its N-terminal tip region and cholesterol interactions. The cytoskeletal elements are involved in the ultrasound sensitivity of hsTRPA1. The finding echoes the hypothesis that N-terminal ankyrin repeats of TRP channels interact with cytoskeletal elements and act as a gating spring in response to mechanical stimuli (Corey et al., 2004; Sotomayor et al., 2005; Christensen and Corey, 2007).

Using ultrasound as the force source avoids the damage from perforating the cell membrane. The precise forces and nanoscale deformations caused by ultrasound combined with multiscale computational modeling and biophysical techniques are superior in mechanobiology (Yoo et al., 2022). Of note, even a variety of ultrasound modalities can be used to dissect the gating mechanism of MS channels; the forms of force loading are usually speculated by computational simulation without further supporting evidence. The use of developed or novel force-sensing reporters could contribute to establishing the gating mechanism by ultrasound stimulation (Meng and Sachs, 2011; Wang et al., 2011; Guo et al., 2014).

Indentation of the PDMS substrate, single pillar bending, and micropipette-guided ultrasound are beneficial tools for investigating the tethering model. The comparison is depicted in Figure 2.

## The Role of ECM in the Force-From-Tether Model

As compared with organisms with a cell wall, in animal cells, the plasma membrane usually directly faces superfluous mechanical stimuli from the surrounding molecules and ECM. The integration of numerous biological and mechanical signals arising from surroundings is required for the correct regulation of cell function *in vivo*. The ECM is a meshed and relatively soft structure, serving as external scaffolding. For the balance of the steady-state forces, in addition to regulating integrin signaling, animal cells may allow the transmission of fast mechanical oscillations *via* direct physical connections between the ECM and MS ion channels. ECM may serve as a device to transmit the force directly or indirectly to open the channel. The investigation of how the ECM interacts with the channel to trigger the mechanotransduction thus becomes important for a better understanding of the tethering model. Although the specialized extracellular components tip links in auditory hair cells are prominent examples for the tethering model, some evidence has identified the specific role of the ECM in the gating mechanism of MS channels.

Studies of the cluster of nematode MEC proteins provide clues to dissecting the tethering model of mammal MS channels. MEC-4 and MEC-10 proteins are attached to a number of intracellular and extracellular MEC proteins to form a touch-sensitive complex (Chalfie, 2009). MEC-1, an ECM protein with multiple epidermal growth factors and Kunitz domains, is required for the accumulation of the collagen MEC-5 and touch sensitivity. MEC-9, another protein needed for touch sensitivity, also contains multiple epidermal growth factor-like and Kunitz-like domains (Emtage et al., 2004). MEC-1 and MEC-5 colocalize with the MS channel complex, but they and MEC-9 are also needed for punctate clustering of the channel components (Emtage et al., 2004). The neuron–epithelial cell interfaces composed of MEC1/5/9 are instrumental in mechanosensory complex assembly and function. However, some evidence shows that the effect of such ECM components on nematode mechanosensitivity is contributed by the direct interaction with the MS channels. For supporting the importance of ECM to mechanosensation, a preprint report implied that somatosensory neurons secrete proteins that actively repurpose the basal lamina to bridge the neuronal plasma membrane to the ECM for vibrotactile sensation (Das et al., 2022). In contrast, several results also showed that modulation of the lipid environment, especially the presence of cholesterol, is important for the function of the MEC mechanotransduction complex (Getz and Reardon, 2004; Huber et al., 2006; Chalfie, 2009). Therefore, Bounoutas and Chalfie proposed the single-tether model; that is, displacement of the lipid bilayer around the channel produces forces that change the conformation state of the channel (Bounoutas and Chalfie, 2007).

In mammal cells, TRPV2 and TRPC1 could be regulated by the dystrophin–glycoprotein complex that connects with the ECM (Vandebrouck et al., 2007; Zanou et al., 2009; Matsumura et al., 2011; Harisseh et al., 2013). In addition, the gating of TRPV2 is facilitated by laminin under the shearing stress of fluid flow in C_2_C_12_ myoblast cells (Kurth et al., 2015), which suggests mechanical forces transmitting to stretch-activated channels by coupling the cytoskeleton with extracellular laminin (Batchelor and Winder, 2006). In mammal neurons, Hu et al. (2010) used transmission electron microscopy to demonstrate that an extracellular tether of about 100 nm synthesized by sensory neurons may link MS channels to the ECM. The ablation of these tether filaments by treating with non-specific and site-specific endopeptidases abolished MS currents in sensory neurons without affecting electrical excitability (Hu et al., 2010). The authors also observed that the MS current may be related to a laminin-containing substrate (Li and Ginty, 2014). In the following study, Chiang et al. (2011) indicated that substrate-bound laminin-332, a major component of keratinocyte-derived ECM, specifically suppressed the rapidly adapting MS current. Li and Ginty (2014) also showed ∼100-nm long extracellular filaments associated with hair follicles in sections prepared from mouse skin. The authors speculated that the tether-like structures at hair follicles may facilitate the excitation of lanceolate endings following hair deflection (Li and Ginty, 2014). As described above, Gaub et al. used atomic force microscopy force measurements to show that cantilevers coated with collagen IV but not EHS laminin or laminin 551 could mechanically stimulate PIEZO1 receptors in living N2a and HEK 293T cells with an increase of Ca^2+^ influx (Gaub and Müller, 2017). Although no further data suggested collagen IV directly interacting with PIEZO1, the authors provide evidence, to some extent, that the gating of PIEZO1 could be mediated by specific ECM-dependent tethering. Such specific laminin-dependent activation was also found in WM-266/4 melanoma cells. Patkunarajah et al. (2020) compared different types of laminin (LM), including LM111, LM211, LM411, and LM511, and observed that LM511-coated pillars gave the most sensitive mechanical current dependent on Elkin1 (TMEM87a) but independent of PIEZO1. All these data reveal that the composition of the ECM can tune the mechanosensitivity of certain MS channels.

After the extracellular tether filaments have been visualized, the next question is whether the filaments directly or indirectly interact with MS channels. Because LMs are the ligands of integrin, the focal adhesion complex is supposed to contribute to the channel tethering. Integrins act as receptors for extracellular proteins, including collagen, laminin, fibronectin, vitronectin, and thrombospondins (Martino et al., 2018), but little is known about the role of integrins in the tethering model. Integrins are well known to mediate focal adhesions, which mechanically connect the ECM to the cytoskeleton and the communication between the ECM and intracellular proteins. However, we lack evidence of the interaction between integrins and MS channels. Ellefsen et al. (2019) provided a model that Myosin II motoring along actin filaments tethered to integrin-based focal adhesion regions generates local increases in membrane tension. PIEZO1 near the vicinity of force-producing adhesions could be activated by the increase in membrane tension. In earlier studies, magnetic manipulation was used to apply mechanical force directly to integrins. The application of a magnetic field to RGD-magnetic beads attached to integrin induced calcium entry in endothelial cells, and this calcium-signaling response could be suppressed by the MS channel blocker gadolinium chloride (Matthews et al., 2006). In addition, Ellefsen et al. (2019) found that PIEZO1 channels are mobile in the cell membrane, and calcium flicker activity is only enriched near force-producing adhesions, which suggests that locally increased membrane tension generated by traction forces can activate PIEZO1 within spatial microdomains. Similarly, Yao et al. (2020) observed that PIEZO1 was recruited to focal adhesions to regulate adhesion maturation in a force-dependent manner. These data suggest that ECM–integrin–cytoskeleton focal adhesion linkages could be important for gating some MS channels (Matthews et al., 2006; Matthews et al., 2007; Martinac, 2014; Ellefsen et al., 2019) and provide a fresh perspective that tethered structure proteins such as integrins and myosin II may transmit forces to MS channels *via* not only auxiliary tethering proteins but also lipid bilayers (Ellefsen et al., 2019).

In terms of the direct connection between MS channels and ECM, Knoepp et al. (2020) discovered that post-translation modification in the extracellular domains of ENaC may be the key to bridging the gaps (Knoepp et al., 2018b; Knoepp et al., 2020). In a series of studies, the group identified that two glycosylated asparagines and their N-glycans, localized in the palm and knuckle domains of αENaC, respectively, are part of tethers to activate endothelial ENaC for shearing force sensing. N-glycans as adhesion molecules can facilitate cell–cell interactions by the interactions of glycan–glycan or glycan–protein. Therefore, the connection with the ECM/glycocalyx *via* N-glycan as anchoring points for tethers may be the principle for the mechanical activation of ASIC channels that share a similar structure with ENaC. Removal of the N-glycosylated asparagines does not completely diminish the response for shearing force. Thus, other mechanisms may contribute to shearing force-induced ENaC activation. Notably, Li et al. (2021) indicated that the status of N-linked glycosylated PIEZO1 is critical for membrane trafficking. The N-glycan of PIEZO1 may interact with other membrane proteins or the ECM molecules, which suggests an extra ECM-modulating mechanism to regulate mechanosensitivity. It also implies that post-translation modification contributing to ECM connection may be a common mechanism vital for bilayer-stretching and filament-tethering gated MS channels.

## The Role of the Cytoskeleton in the Mechanogating of Mechanosensitive Ion Channels

The cytoskeleton maintains the cell’s shape, builds a functional and a structural compartment of cells, and modulates multiple pathways of cellular mechanotransduction. The cortical cytoskeleton is anchored to the lipid bilayer *via* dynamic interactions between lipids, ankyrin, and integral membrane proteins. The tension of the cytoskeleton network is transmitted *via* the cell cortex and greatly influences the gating of MS ion channels. The cytoskeleton plays roles in the model of the force-from-filament and also the model of the force-from-lipids. For instance, PIEZO1 is activated by bilayer tension in membrane blebs lacking the cortical cytoskeleton. The mechanoprotection by the cytoskeleton in the classical cell-attached patch recordings confers PIEZO1 gating at higher pressure (Cox et al., 2016). This finding is consistent with a report indicating that knockout of filamin A, a scaffold protein that connects the actin network to membrane proteins, decreased the activation threshold of PIEZO1 in cell-attached patch clamp assays (Retailleau et al., 2015). Another report showed that filamin A is also involved in the Polycystin-1/2 (TRPP1/2)-mediated mechanosensitivity of stretch-activated ion channels (Sharif-Naeini et al., 2009). The authors demonstrated that the ratio of TRPP1 to TRPP2 controls the mechanosensitivity *via* filamin A coupled to the actin cytoskeleton and affects the conversion of intraluminal pressure to local bilayer tension (Sharif-Naeini et al., 2009). In addition, the mechanosensitivity of TREK-1, a K_2P_ channel that is highly expressed in fetal neurons, was reported to be tonically repressed by the actin cytoskeleton (Lauritzen et al., 2005). In contrast, the mechanogating function of PIEZO2 was influenced greatly by *in vitro* treatment with latrunculin A, an actin-depolymerizing toxin, as compared with PIEZO1, which was less affected by latrunculin A (Eijkelkamp et al., 2013). The microtubule-depolymerizing agent colchicine also blunted PIEZO2 mechanosensitivity by reducing the peak currents. These findings imply that PIEZO2 applies actin and tubulin to the mechanogating *via* distinct mechanisms (Eijkelkamp et al., 2013). Other studies of chemical destruction of the cytoskeleton also indicated that the cytoskeleton has more impact on PIEZO2 than PIEZO1 mechanogating (Romero et al., 2019; Romero et al., 2020). Even though several lines of functional assays implicated the cytoskeleton in the gating of PIEZO2, there is still no biochemical evidence of a direct connection between PIEZO2 and the cytoskeleton. Furthermore, in contrast with NompC, possessing ankyrin repeats for the microtubule linkage, PIEZO2 has less structural clues for the cytoskeleton binding. Nonetheless, a study presented a functional link between the PIEZO2 beam domain and the cytoskeleton. Romero et al. (2020) engineered a PIEZO2 chimera with the intracellular beam domain replaced by that of PIEZO1. The PIEZO2-PIEZO1 beam chimera lost much of its sensitivity to latrunculin A. Verkest et al. (2022) reported that an intrinsically disordered domain 5 (IDR5) in the PIEZO2 molecule was required for the interaction between PIEZO2 and the actin cytoskeleton. Deletion of IDR5 specifically reduced membrane indentation-evoked PIEZO2 currents, but showed no effects on negative pressure-evoked PIEZO2 currents. The actin cytoskeleton-disrupting drug cytochalasin-D also reduced membrane indentation-evoked current amplitudes of PIEZO2 to the levels of the IDR5-deleted mutant. The authors showed that IDR5 is required for detecting cell-generated forces that activate PIEZO2 *via* force-from-filament or force-from-lipids-*via*-filament, but not fore-from-lipids (Verkest et al., 2022). In addition, the presence of some cytoskeletal proteins, such as STOML3, increased the mechanosensitivity of both PIEZO1 and PIEZO2 to membrane tension (Poole et al., 2014). The roughness-dependent sensitization of PIEZO1 was ablated by disrupting the actin cytoskeleton or myosin II-dependent cellular contractility (Bavi et al., 2019). The relationship between the cellular cytoskeleton and PIEZO1 activation is covered in the review by Nourse and Pathak (Nourse and Pathak, 2017).

Of note, the lack of channel response to damaging the cytoskeleton network is not proof of an MS channel merely gated by the force-from-filament. Membrane-bound cytoskeleton is considered to act as a tonic suppressor to limit channel activity induced by membrane stretch in the experiments of patch excision, and this process is called mechanoprotection (Patel et al., 1998; Patel and Honoré, 2001; Honoré et al., 2006). Disrupting the cytoskeleton exposes the lipid bilayers to a larger fraction of the applied force. Furthermore, Shi et al. (2018) indicated that propagation of the membrane tension equilibrates faster in cell-attached blebs without a cytoskeleton but slower in cells with an intact cortical cytoskeleton, which suggests that the cytoskeleton may increase local membrane tension. Hence, living cells behave differently from the reconstituted membranes, with the cytoskeleton suppressing the equilibration of tension over the whole membrane structure. All these observations suggest that the cortical cytoskeleton and the cell membrane should be considered a composite material, not two separate structures.

In the aspect of the tethering model, NompC is an example depicting the tether-gated mechanism through the microtubule cytoskeleton (Jin et al., 2020). Structural and functional studies have shown that NompC physically interacts with microtubules and that the microtubule is required for NompC activation (Jin et al., 2020). NompC is colocalized with microtubules in the heterologous expression system (Cheng et al., 2010; Yan et al., 2018). The amino terminus of NompC has 23 ankyrin repeat domains that are proposed to function as a flexible linker to the cytoskeleton (Zhang et al., 2015; Jin et al., 2020). Disruption of microtubules inhibits NompC-dependent mechanosensing in *Drosophila* (Suchyna et al., 2009; Liang et al., 2013). In contrast, stabilizing or disrupting the actin filaments had no effect on NompC mechanogating (Zhang et al., 2015). Membrane–microtubule connectors of *Drosophila* are absent in nompC mutants and were rescued by nompC gene overexpression (Liang et al., 2013). In contrast, the role of microtubules in the MEC-4/10-mediated mechanotransduction is debated. The specialized microtubules in the touch receptive neurons of *C. elegans* influence MEC-4/10-mediated mechanotransduction (Chalfie and Sulston, 1981). However, O'Hagan et al. (2005) reported the tethering to 15-protofilament microtubules is not required for mechanogating of the MEC-4 channel complex, although microtubules are needed for normal sensitivity to mechanical stimulation.

## Conclusion

Each of Aristotle’s five senses almost corresponded to a discernible sense organ in the body. However, mechanosensation, the fundamental mechanism of touch, exists in every cell. The sense of temperature, which Aristotle folds into touch, is also critical for every organ in our body. The detection of force and heat may be intertwined with each other in biology. When thermal energy induces the rearrangement of the lipid bilayer, it may alter membrane tension to gate the polymodal ion channels (Clapham, 2003; Kung, 2005). However, we lack robust evidence to show that polymodal channels such as TRP channels can be gated purely by the stretching of lipid bilayers (Martinac and Cox, 2017). The question is how cells distinguish between touch and heat. Some receptors may perceive heat and force to sum up as pain. Others develop distinct modalities in addition to the model of stimulus-from-lipid, such as intrinsic high thermosensitivity for heat sensation and gating by filament stretching for force sensation (Zheng, 2013). Because TRP channels are constructed to possess an unusually high Q10 value (range 10–27), some TRP members and ASICs use the complicated network of ECM-bilayer/channel-cytoskeleton to detect the stimuli of force change. Apart from merely responding to osmotic forces, mammal cells, especially neuron cells, have evolved to develop a complicated system with multiple modalities to deal with the variety of environmental stimuli. Different tissues receive distinct forms of mechanical force. The contribution of the cytoskeleton in mechanotransduction by PIEZO1 is lower than the contribution of bilayer tension. In contrast, mechanotransduction mediated *via* somatosensory PIEZO2 may include cytoskeleton, scaffold proteins, and ECM to deal with complicated mechanical stimulations. Under physiologic conditions, how a tethering force transmits the stress to gate the MS channel open is an important issue to address. In addition to the paradigm of force-from-filament, the model of force sharing may give more comprehensive insight into the role of the cytoskeleton, ECM, and MS channel-auxiliary components in MS channel modulation (Cox et al., 2019). New approaches and new models of biophysics are required to solve the unanswered questions. Better understanding the exact roles of mechanosensors and their functional mechanism will help elucidate mechanical-related human diseases and inspire us to establish new strategies for disease therapies.
